# Isolation of group B *Streptococcus* with reduced *β*-lactam susceptibility from pregnant women

**DOI:** 10.1080/22221751.2018.1557987

**Published:** 2019-01-16

**Authors:** Hiroaki Moroi, Kouji Kimura, Tomomi Kotani, Hiroyuki Tsuda, Hirotsugu Banno, Wanchun Jin, Jun-ichi Wachino, Keiko Yamada, Takashi Mitsui, Mamoru Yamashita, Fumitaka Kikkawa, Yoshichika Arakawa

**Affiliations:** a Department of Bacteriology, Nagoya University Graduate School of Medicine, Nagoya, Japan; b Department of Obstetrics and Gynecology, Nagoya University Graduate School of Medicine, Nagoya, Japan; c Kishokai Medical Corporation, Inazawa, Japan

**Keywords:** Group B *Streptococcus*, GBS, *Streptococcus agalactiae*, pregnant women, reduced *β*-lactam susceptibility

## Abstract

*β*-Lactam antibiotics are first-line agents for the treatment and prevention of group B *Streptococcus* (GBS) infections. We previously reported clinical GBS isolates with reduced *β*-lactam susceptibility (GBS-RBS) and characterized them as harbouring amino acid substitutions in penicillin-binding proteins (PBPs). However, to our knowledge, GBS-RBS clinical isolates have never previously been isolated from pregnant women worldwide. We obtained 477 clinical GBS isolates from vaginal/rectal swabs of 4530 pregnant women in Japan. We determined the MICs of seven *β*-lactams for all 477 clinical isolates. Five clinical isolates showed reduced ceftibuten susceptibility. For these isolates, we performed sequencing analysis of *pbp* genes. None of the 477 isolates were non-susceptible to penicillin G, ampicillin, and meropenem. For five isolates, the MICs of ceftibuten were relatively high (64–128 μg/ml). Each of these isolates possessed a single amino acid substitution in PBP2X, and some of the substitutions had been previously found in GBS with reduced penicillin susceptibility. This is the first report of the isolation of clinical GBS-RBS isolates harbouring amino acid substitutions in PBP2X that confer reduced ceftibuten susceptibility from pregnant women.

## Introduction

Group B *Streptococcus* (GBS; *Streptococcus agalactiae*) is a gram-positive bacterium that causes neonatal invasive infections such as sepsis and meningitis. Neonatal GBS infections are classified into two types, early-onset disease (first six days of life) and late-onset disease (first 7–90 days of life), and it is considered that early-onset disease can be caused by vertical transmission from GBS-colonized mothers during delivery [1]. The Centers for Disease Control and Prevention (CDC) issued guidelines for the prevention of perinatal GBS disease in 1996 [2]. In these guidelines, universal screening for GBS of all pregnant women at 35–37 weeks of gestation and intrapartum antimicrobial prophylaxis for GBS-positive pregnant women are recommended to prevent vertical GBS transmission. The guidelines were updated in 2002 and 2010 [2]. The Japanese Society of Obstetrics and Gynecology (JSOG) also published “Guidelines for Obstetrical Practice in Japan” in 2007 (revised in 2011 and 2014), in which they recommend universal screening for GBS of all pregnant women at 33–37 weeks of gestation for the prevention of vertical GBS transmission nationwide [3].

Because all clinical GBS isolates are regarded to be fully susceptible to *β*-lactam antibiotics, including penicillin G, *β*-lactam antibiotics are the first-line agents for the treatment and prevention of GBS infections [2,4]. However, in 2008, we reported the isolation of clinical GBS strains with reduced penicillin susceptibility (PRGBS) and their molecular characterization [5]. Since our first report of PRGBS, similar clinical PRGBS isolates have been reported by other groups from the USA, Canada, and Japan [6–10]. Moreover, penicillin-susceptible GBS isolates with reduced cephalosporin susceptibility (CTB^r^PSGBS) were identified in Japan in 2014 [11], and the identification of penicillin-susceptible GBS with reduced cefotiam susceptibility was also reported in Japan [12]. We denoted these clinical isolates as GBS with reduced *β*-lactam susceptibility (GBS-RBS) [13]. Clinical GBS-RBS isolates acquire amino acid substitutions in the transpeptidase domain of penicillin-binding proteins (PBPs), especially PBP2X, which is one of the target molecules of *β*-lactams. Although several amino acid substitutions in PBP2X associated with reduced *β*-lactam susceptibility have been identified, most clinical PRGBS isolates possess V405A and/or Q557E amino acid substitutions in PBP2X (Supplementary Figure 1). Clinical PRGBS isolates are non-susceptible to oxacillin, ceftizoxime, and ceftibuten in addition to penicillin G [5,14]. We recently reported that the isolation rate of PRGBS increased from 2.3% to 14.7% between 2005–2006 and 2012–2013 [15]. To our knowledge, clinical GBS-RBS isolates confirmed to harbour amino acid substitutions in PBPs have never been isolated from pregnant women worldwide [16]. However, considering the increasing isolation rate of PRGBS, it is possible that GBS-RBS isolates have been detected in pregnant women. In this study, through the screening of 4530 vaginal/rectal specimens from pregnant women in Japan, we obtained 477 GBS clinical isolates and analysed whether or not GBS-RBS isolates could be recovered from pregnant women.

## Results

### Determination of MICs

We determined the MICs of nine antibiotics for the 477 isolates by the agar dilution method (Table 1). According to CLSI criteria, none of the isolates were non-susceptible to penicillin G, ampicillin, and meropenem, 153 isolates (153/477, 32.1%) were non-susceptible to erythromycin, and 60 isolates (60/477, 12.6%) were non-susceptible to clindamycin. For five clinical isolates, the MICs of ceftibuten were relatively high (64–128 μg/ml), and for two of these isolates, the MIC of ceftizoxime was also high (4 μg/ml) (Table 2). These findings suggested that these five clinical isolates might be GBS-RBS clinical isolates harbouring amino acid substitutions in PBPs. Of these five isolates, three were resistant and one was intermediate to erythromycin.

**Table 1.** Distributions of MIC for 477 GBS isolates from pregnant women.

Antibiotics	MIC (μg/ml)															Number of non-susceptible isolates (%)
	≤0.01	0.03	0.06	0.12	0.25	0.5	1	2	4	8	16	32	64	128	>128	
Penicillin G	7	264	204	2												0 (0)
Ampicillin	1	18	160	276	22											0 (0)
Oxacillin			2	7	248	218	2									*
Cefazolin			25	248	182	22										*
Ceftibuten									6	110	322	34	2	3		*
Ceftizoxime			14	282	172	3	2	2	2							*
Meropenem	8	328	132	9												0 (0)
Erythromycin			20	192	82	30	33	28	20	10	5	1	2	3	51	153 (32.1)
Clindamycin		7	145	244	16	5	3	1	6	1	0	3	3	4	39	60 (12.6)

*The Clinical and Laboratory Standards Institute (CLSI) does not provide breakpoints of oxacillin, cefazolin, ceftibuten, and ceftizoxime.

The breakpoints for categorisation as “Susceptible” set by the CLSI are ≤0.12 μg/ml (penicillin G), ≤0.25 μg/ml (ampicillin), ≤0.5 μg/ml (meropenem), ≤0.25 μg/ml (erythromycin), and ≤0.25 μg/ml (clindamycin).

Abbreviation: GBS, group B *Streptococcus*; MIC, minimum inhibitory concentration.

**Table 2.** MICs of antimicrobial drugs for GBS isolates with reduced cephalosporin susceptibility.

Isolate	MIC (μg/ml)								
	PEN	AMP	OXA	CFZ	CTB	ZOX	MEM	ERY	CLI
P-071	0.06	0.12	0.5	0.25	128*	2	0.06	0.12	0.12
P-122	0.06	0.12	0.5	0.5	256*	4	0.03	2	0.12
P-139	0.03	0.06	0.5	0.5	256*	4	<0.015	>128	0.25
P-319	0.03	0.12	0.5	0.12	64*	1	0.03	1	0.12
P-334	0.03	0.12	0.5	0.12	128*	1	0.06	0.5	0.12

Abbreviations: MIC, minimum inhibitory concentration; GBS, group B *Streptococcus*; PEN, penicillin G; AMP, ampicillin; OXA, oxacillin; CFZ, cefazolin; CTB, ceftibuten; ZOX, ceftizoxime; MEM, meropenem; ERY, erythromycin; CLI, clindamycin

### Sequence analysis of *pbp* genes

**Figure 1.**
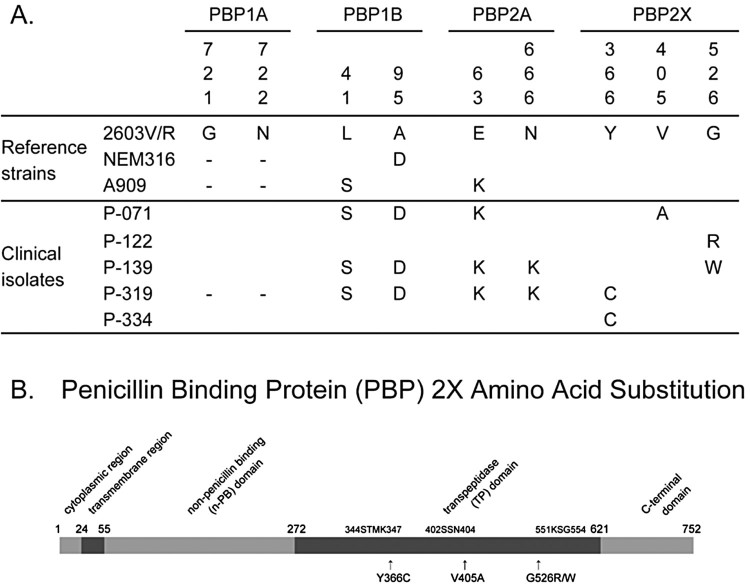
(A) Amino acid sequences of penicillin binding proteins (PBPs) of control strains (2603V/R, NEM316, A909) and clinical isolates with reduced ceftibuten susceptibility recovered from pregnant women. The position of each amino acid is indicated above the sequence. “–” indicates an amino acid deletion. Amino acid substitutions found in PBP1A, 1B, and 2A of clinical isolates in the present study were previously found in penicillin-susceptible group B *Streptococcus*. We did not find any amino acid substitutions of PBP2B in clinical isolates. (B) PBP2X diagram and amino acid substitutions. The five GBS-RBS isolates in this investigation possessed single amino acid substitutions near the active-site motifs of the transpeptidase domain of PBP2X (Y366C, V405A, and G526R/W).

**Figure 2.**
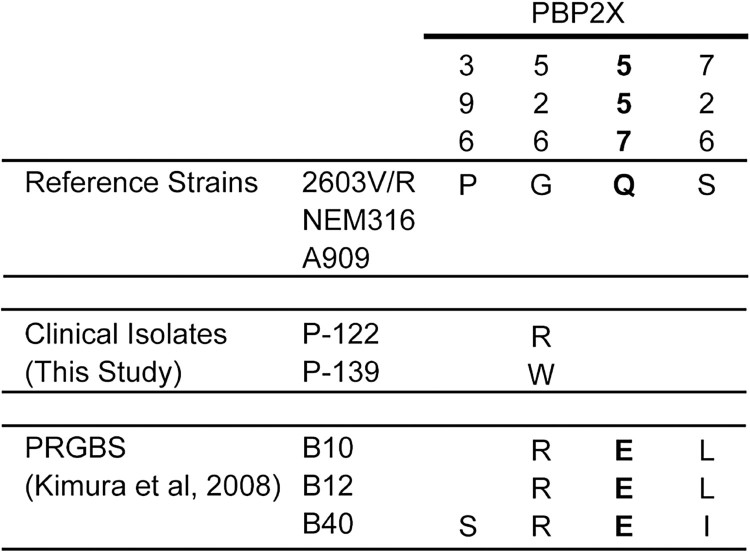
Amino acid substitutions in PBP2X among clinical group B *Streptococcus* with reduced *β*-lactam susceptibility (GBS-RBS) isolates in this investigation and previously reported PRGBS isolates. Two GBS-RBS clinical isolates, P-071 and P-122, possessed single amino acid substitutions that were found in PBP2X of clinical PRGBS isolates. Should the clinical isolate P-122 acquire Q557E and S726L in PBP2X in future, the amino acid sequence of its PBP2X would be identical to those in the previously reported clinical PRGBS isolates B10 and B12.

We conducted sequence analysis of the *pbp* genes in each of the five GBS isolates with reduced ceftibuten susceptibility (Figure 1). Each isolate possessed a single amino acid substitution in the deduced amino acid sequence of PBP2X. One clinical isolate, P-071, carried a V405A amino acid substitution, which is commonly detected in PBP2X in PRGBS (Supplementary Figure 1). The clinical isolate P-122 possessed a G526R substitution that has been found in PRGBS previously (Figures 1 and 2). The G526W substitution in PBP2X of clinical isolate P-139 and the Y366C substitution in PBP2X of isolates P-319 and P-344 have never before been reported. Although amino acid substitutions and deletions in PBP 1A, PBP 1B, and PBP 2A were detected in a few of the five isolates, these mutations have been reported in GBS isolates that do not show reduced *β*-lactam susceptibility and were therefore considered not to be responsible for the reduced *β*-lactam susceptibility.

### Allelic-exchange strains

To reveal the contributions of the amino acid substitutions in PBP2X of the clinical isolates P-071, P-122, P-139, P-319, and P-334 to their reduced *β*-lactam susceptibility, we generated four allelic-exchange strains, BAA-611 (P-071 PBP2X), BAA-611 (P-122 PBP2X), BAA-611 (P-139 PBP2X), and BAA-611 (P-319/P-334 PBP2X). In comparison with the corresponding MICs for BAA-611, the MICs of ceftibuten and ceftizoxime for the allelic-exchange strains were elevated and were comparable to those for the parental clinical isolates, P-071, P-122, P-139, P-319, and P-334 (Table 3). These results indicated that amino acid substitutions in PBP2X of the five GBS isolates with reduced ceftibuten susceptibility are responsible for their reduced *β*-lactam susceptibility, and especially, their reduced ceftibuten susceptibility.

**Table 3.** MICs of β-lactams for allelic-exchange strains.

Strain/isolate	MIC (μg/ml)						
	PEN	AMP	OXA	CFZ	CTB	ZOX	MEM
BAA-611	0.03	0.12	0.5	0.25	16	0.5	0.06
BAA-611(P-071 PBP2X)	0.06	0.12	1	0.5	256	4	0.06
BAA-611(P-122 PBP2X)	0.06	0.12	1	1	256	8	0.06
BAA-611(P-139 PBP2X)	0.03	0.12	0.5	0.5	>256	8	0.03
BAA-611(P-319/P-334 PBP2X)	0.06	0.12	0.5	0.25	256	4	0.12
P-071	0.06	0.12	1	0.25	256	4	0.06
P-122	0.06	0.12	1	1	256	8	0.06
P-139	0.03	0.12	0.5	1	>256	8	0.03
P-319	0.06	0.12	1	0.25	>256	4	0.12
P-334	0.06	0.12	1	0.25	256	4	0.12

Abbreviations: MIC, minimum inhibitory concentration; PEN, penicillin G; AMP, ampicillin; OXA, oxacillin; CFZ, cefazolin; CTB, ceftibuten; ZOX, ceftizoxime; MEM, meropenem

### Genetic background of clinical isolates with reduced ceftibuten susceptibility

Sequence types of the five GBS isolates showing reduced ceftibuten susceptibility were determined by multilocus sequence typing (MLST) (Table 4). Two isolates belonged to ST1, another two to ST335, and the fifth to ST41. eBURST analysis revealed that ST1, ST335, and ST41 were not closely related to one another (Supplementary Figure 2). ST1 and ST335 belonged to CC1 and CC19, respectively, and the most closely related CC of ST41 was CC10. The isolates P-139 and P-319 both belonged to ST1, whereas P-122 and P-334 both belonged to ST335. However, despite these isolates being paired by sequence type, they did not share identical serotypes, implying that they might be non-clonal (Table 4).

**Table 4.** Origins, genetic backgrounds, and clinical information of five GBS-RBS isolates.

Isolate	Institute	Serotype	ST	Method of delivery	Reason for CS	Antibiotics during intrapartum period	Maternal infection	Neonatal infection
P-071	A	II	41	CS	CPD	CTM	–	–
P-122	B	IV	335	TVD		AMP	–	–
P-139	B	NT	1	TVD		AMP	–	–
P-319	C	III	1	CS	Arrested labour	AMP, CTM	–	–
P-334	D	III	335	TVD		AMP	–	–

Abbreviations: GBS-RBS; group B *Streptococcus* with reduced β-lactam susceptibility; ST, sequence type; CS, caesarean section; NT, non-typeable; TVD, transvaginal delivery; CPD, cephalopelvic disproportion; CTM, cefotiam; AMP, ampicillin

### Origins and clinical outcomes

The origins of the five GBS isolates with reduced ceftibuten susceptibility and perinatal outcomes are shown in Table 4. Two women received prenatal check-ups at institute B, whereas the other women from whom the isolates were recovered had been monitored at different institutes. P-122 and P-139 were both recovered from institute B; however, the women from whom these isolates were recovered were screened for GBS at different times. Three of these five women delivered their children by transvaginal delivery, and two by caesarean section. Medical reasons for caesarean section were cephalopelvic disproportion and arrested labour. Ampicillin was used as the prophylactic antibiotic within the guidelines in the three cases of transvaginal delivery. In the two caesarean section cases, cefotiam was used to prevent postoperative infection. No episode of active GBS infection was seen in the mothers or neonates during the perinatal period.

## Discussion

In this investigation, we recovered five clinical isolates from pregnant women that showed reduced ceftibuten susceptibility. Since our first report of PRGBS in 2008 [5], we have reported several studies on GBS-RBS in Japan. Clinical GBS-RBS isolates, including PRGBS isolates, have been recovered from respiratory specimens, urine, blood, joint fluid, or other samples from non-pregnant adults, and most frequently from the elderly. Numerous reports have described nationwide or regional studies on the antimicrobial susceptibility of clinical GBS isolates recovered from pregnant women. However, to our knowledge, clinical GBS-RBS isolates harbouring amino acid substitutions in PBPs had not been recovered from pregnant women [16]. The five GBS-RBS isolates detected in this investigation were recovered from four geographically separated institutes. The two clinical isolates recovered from the same institute were isolated at different times and had different serotypes and sequence types (Table 4), suggesting that the five GBS-RBS isolates detected in this investigation were not clonal. Therefore, this is the first report worldwide of five non-clonal clinical GBS-RBS isolates harbouring amino acid substitutions in PBPs from pregnant women.

Each of the five clinical GBS-RBS isolates possessed a single amino acid substitution in PBP2X. The substitutions in isolates P-071 and P-122 have also been found in clinical PRGBS isolates. Clinical PRGBS isolates typically harbour three to seven amino acid substitutions in PBP2X. Should isolate P-122 acquire Q557E and S726L in PBP2X in future, the amino acid sequence of its PBP2X protein would be identical to those of the previously reported clinical PRGBS isolates B10 and B12 (Figure 2). This might imply that the risk of PRGBS emergence among pregnant women is increasing.

Certainly, ceftibuten is not recommended as an antimicrobial agent for the prevention of perinatal GBS infections, and GBS-RBS are rare in pregnant women (5/477, 1.0%) at present, as shown by this study. For the GBS-RBS isolates studied in this investigation, our data suggested that ampicillin may be effective as a prophylactic agent in the intrapartum period. Therefore, the detection of GBS-RBS from pregnant women in this investigation does not indicate an increased direct risk of neonatal GBS disease. However, our previous study revealed that the isolation rate of PRGBS increased from 2.3% to 14.7% between 2005–2006 and 2012–2013 [15]. Moreover, multidrug-resistant PRGBS (MDR-PRGBS), which is non-susceptible to macrolides and fluoroquinolones in addition to penicillins, accounted for 68.9% of clinical PRGBS isolates in 2012–2013 [15]. PRGBS/MDR-PRGBS strains have been isolated mainly from non-pregnant adults and most frequently from the elderly. However, a general increase in the prevalence of clinical MDR-PRGBS isolates might be a risk factor for the emergence of MDR-PRGBS among pregnant women. Moreover, we previously reported that one clinical PRGBS isolate showed high ceftizoxime resistance due to amino acid substitutions in PBP1A in addition to PBP2X [17]. If the MICs of *β*-lactams, especially penicillins, for GBS isolates from pregnant women increase due to additional amino acid substitutions in PBPs or transmigration of PRGBS, the prevention strategy for perinatal GBS infections might have to be adjusted.

In summary, this is the first report of the isolation of clinical GBS-RBS isolates from pregnant women. The MICs of ceftibuten were high for these isolates, which possessed single amino acid substitutions in the transpeptidase domain of PBP2X. Although the isolates were susceptible to penicillin G and ampicillin, careful monitoring of *β*-lactam susceptibility among GBS isolates from pregnant women will be needed. Considering that GBS strains show high rates of erythromycin and clindamycin resistance, drug susceptibility testing of GBS isolates from pregnant women with penicillin allergy is crucial for drug choice. Furthermore, drug susceptibility testing of all GBS isolates from pregnant women might be advisable for the careful monitoring of GBS drug resistance.

## Materials and methods

### Determination of MICs

We determined the MICs of nine antimicrobial drugs (penicillin G, ampicillin, oxacillin, cefazolin, ceftibuten, ceftizoxime, meropenem, erythromycin, and clindamycin) for the clinical GBS isolates by the agar dilution method, as recommended by the CLSI [4]. The CLSI does not provide breakpoints of oxacillin, cefazolin, ceftibuten, and ceftizoxime for GBS. The strain *Streptococcus pneumoniae* ATCC 49619 was used as a quality control.

### Sequence analysis of *pbp* genes

We conducted sequence analyses of the five genes encoding PBPs (PBP1A, PBP1B, PBP2A, PBP2B, and PBP2X) in five GBS isolates that showed reduced *β*-lactam susceptibility. PCR amplification and sequencing were carried out as previously described [5]. Each sequence was compared with the reference sequence of GBS strain ATCC BAA-611/2603V/R (GenBank accession no.: NC_004116). The DNA Data bank of Japan (DDBJ) accession numbers of PBP genes in clinical isolates P-071, P-122, P-139, P-319, and P-334 are LC372501–LC372525.

### Generation of allelic-exchange strains

Using strain *S. agalactiae* ATCC BAA-611 and the thermosensitive shuttle vector pG + host6Δamp, we generated four allelic-exchange strains harbouring amino acid substitutions in PBP2X that are identical to those in clinical isolates P-071, P-122, P-139, and P-319/P-334, respectively, as previously reported [5], with minor modifications. Double cross-over strains were selected on Todd–Hewitt agar plates containing 64 μg/ml of ceftibuten. We designated the allelic-exchange strain harbouring an amino acid substitution in PBP2X identical to that of clinical isolate P-071 as BAA-611(P-071 PBP2X). Similarly, we designated the other strains as BAA-611(P-122 PBP2X), BAA-611(P-139 PBP2X), and BAA-611(P-319/P-334 PBP2X). It was confirmed that there was no additional nucleotide change in the *PBP2X* gene of the generated allelic-exchange strains by Sanger sequencing. MICs for the allelic-exchange strains were determined by the agar dilution method as described above.

### MLST analysis

MLST analysis was performed for clinical GBS isolates that showed reduced *β*-lactam susceptibility. We PCR-amplified seven partial housekeeping genes (*adhP*, *pheS*, *atr*, *glnA*, *sdhA*, *blcK*, and *tkt*) and sequenced them. STs of these isolates were determined as described previously [5,18]. The ST and clonal complex (CC) of each isolate was determined using GBS MLST databases (http://pubmlst.org/sagalactiae/) and eBURST V3 (http://eburst.mlst.net/v3/enter_data/single/).

## Ethics statement

This observational study was approved by the research ethics committee of the Nagoya University Graduate School of Medicine and Kishokai Corporation.

## Supplementary Material

Supplemental Material
