# Supplementary material for: Isolation of group B Streptococcus with reduced β-lactam susceptibility from pregnant women
Source: Emerg Microbes Infect. 2019 Jan 16;8(1):2–7. doi: 10.1080/22221751.2018.1557987 (PMC6455180; doi:10.1080/22221751.2018.1557987)
Supplement: Supplemental Material [file TEMI_A_1557987_SM4924.docx]

**Supplementary text**

**Results**

**Confirmation of GBS**

In total, 4,530 pregnant women at 33–37 weeks of gestation were screened at four institutions during the study period. Among the screened subjects, 506 were judged as GBS-positive (11.2%) (Supplementary Table 1). Of these, 477 isolates were confirmed as being GBS at our laboratory by colony formation assay on GBS selective agar plates, β-haemolysis on sheep blood agar plates, and specific agglutination with anti-Lancefield B antigen serum. The remaining 29 isolates could not be recovered at our laboratory after transportation.

**Serotype distribution**

Serotypes of collected GBS isolates were analysed by agglutination test and multiplex PCR. The serotype distribution was shown in Supplementary Table 2. The most dominant serotype was III (24.9%), followed by V, Ia, and Ib (17.2%, 16.8%, and 14.0%). Serotypes VI, II, VIII, IV, and VII were less common (9.4%, 6.9%, 2.5%, 2.3%, and 0.6%, respectively). Twenty-five isolates were non-typeable (5.2%).

**Discussion**

In this investigation, we revealed four patterns of single amino acid substitutions in PBP2X among five GBS-RBS clinical isolates that we recovered from pregnant women. As we reported in our previous studies, most PRGBS isolates harbour several amino acid substitutions in PBP2X, with V405A and/or Q557E being the most common (Supplementary Figure 1). On the other hand, there is only one publication on the recovery of clinical CTB^r^PSGBS isolates at one institute, and those isolates harboured one or two amino acid substitutions, T394A and G429S, in PBP2X, but they did not harbour V405A or Q557E (Supplementary Figure 1)^11^. Interestingly, although the five GBS-RBS clinical isolates recovered from pregnant women in this study showed similar antimicrobial susceptibility profiles to that of previously reported CTB^r^PSGBS, i.e., being penicillin-susceptible and having reduced ceftibuten susceptibility, the amino acid substitutions in PBPs differed among the isolates (Figure 1A and Supplementary Figure 1). The clinical isolate P-071 possessed only one amino acid substitution in PBP2X, V405A, which is characteristic in PRGBS. The clinical isolate P-122 had one amino acid substitution in PBP2X, the G526R substitution, which has also been found in PRGBS clinical isolates (Supplementary Figure 1). The other three clinical isolates possessed single amino acid substitutions, G526W or Y366C, which have not been reported to date. All amino acid substitutions in PBP2X found in this investigation were located near active-site motifs of the transpeptidase domain, the binding site of β-lactams, in PBP2X (Figure 1B). These mutations were not found in PBP2X of GBS, except for the clinical GBS-RBS isolates reported previously, and the allelic-exchange strains harbouring amino acid substitutions in PBP2X identical to those of the five GBS-RBS isolates showed reduced ceftibuten susceptibility. Therefore, these amino acid substitutions in PBP2X of the five GBS-RBS isolates studied in this investigation are the main contributors to reduced β-lactam susceptibility.

According to the MLST analysis, the five GBS-RBS isolates belonged to ST1, ST41, and ST335. In a recent study, ST458 and ST1, belonging to CC1, were the predominant STs among PRGBS in Japan^S1^. The other PRGBS isolates were characterised as ST4 (CC1), ST23 (CC23), and ST464 (CC23)^11, S1^. PRGBS isolates recovered in the USA belonged to ST19, ST22, and ST1^6,7^. Nagano et al. reported that isolates of CTB^r^PSGBS were assigned to ST1^11^. These results suggest that clinical GBS isolates having various genetic backgrounds could become non-susceptible to β-lactams.

**Materials and methods**

**Clinical isolates and identification of GBS**

From September 2014 to September 2015, 4,530 pregnant women attended the Nagoya University Hospital (tertiary perinatal care centre) and 8 primary maternal care providers affiliated to the Kishokai Medical Corporation in the Aichi and Gifu prefectures of Japan. Vaginal/rectal swabs were used for GBS screening between the 33rd and 37th weeks of gestation, following the Japanese guidelines mentioned above. All clinical isolates obtained from these swabs were transferred to our laboratory and were confirmed as GBS by colony formation on CHROMagar™ strepB GBS selective agar plates (Kanto Chemical Co. Inc., Tokyo, Japan), β-haemolysis on sheep blood agar plates, and specific agglutination with anti-Lancefield B antigen serum (Prolex™ streptococcal grouping kit, Iwaki & Co. Ltd., Tokyo, Japan). We obtained one clinical isolate from each pregnant woman.

**Serotyping and molecular capsular typing**

Serotyping of each GBS was conducted with slide agglutination tests with GBS serotype-specific anti-serum (Denka Seiken, Tokyo, Japan). Multiplex PCR assay for capsular typing was carried out for isolates that were non-typeable by agglutination tests, using the primers previously reported by Poyart et al.^S2^. Samples that were non-typeable by both methods were confirmed as GBS by PCR amplification using GBS *dltS*-specific primers^S2^.

**References**

S1 Kimura, K. et al. Predominance of sequence type 1 group with serotype VI among group B streptococci with reduced penicillin susceptibility identiﬁed in Japan. *J. Antimicrob. Chemother.* **66,** 2460–2464 (2011).

S2 Poyart, C. et al. Multiplex PCR assay for rapid and accurate capsular typing of group B streptococci. *J. Clin. Microbiol.* **45,** 1985–1988 (2007).
